# Treatment of Cheyne–Stokes respiration with adaptive servoventilation—analysis of patients with regard to therapy restriction

**DOI:** 10.1007/s11818-020-00269-2

**Published:** 2020-10-08

**Authors:** Sven Stieglitz, Wolfgang Galetke, Antonio Esquinas

**Affiliations:** 1 Department of Pneumology, Allergy, Sleep and Intensive Care Medicine, Petrus Hospital Wuppertal, University of Witten-Herdecke, Carnaper Str. 48, 42283 Wuppertal, Germany; 2 Klinik für Pneumologie, VAMED Klinik Hagen Ambrock, Hagen, Germany; 3 Intensive Care Unit, Hospital Morales Meseguer, Avda, Marqués de los Velez, s/n, 30008 Murcia, Spain

**Keywords:** Central sleep apnoea, Heart failure, Periodic breathing, Sleep apnoea, Cardiorespiratory interaction, Zentrale Schlafapnoe, Herzinsuffizienz, Periodische Atmung, Schlafapnoe, Kardiorespiratorische Interaktion

## Abstract

**Purpose:**

The SERVE-HF study revealed no benefit of adaptive servoventilation (ASV) versus guideline-based medical treatment in patients with symptomatic heart failure, an ejection fraction (EF) ≤45% and a predominance of central events (apnoea-hypopnea Index [AHI] > 15/h). Because both all-cause and cardiovascular mortality were higher in the ASV group, an EF ≤ 45% in combination with AHI 15/h, central apnoea-hyponoea index [CAHI/AHI] > 50% and central apnoea index [CAI] > 10/h were subsequently listed as contraindications for ASV. The intention of our study was to analyse the clinical relevance of this limitation.

**Methods:**

Data were analysed retrospectively for patients treated with ASV who received follow-up echocardiography to identify contraindications for ASV.

**Results:**

Echocardiography was conducted in 23 patients. The echocardiogram was normal in 10 cases, a left ventricular hypertrophy with normal EF was found in 8 patients, there was an EF 45–50% in 2 cases and a valvular aortic stenosis (grade II) with normal EF was found in 1 case. EF <45% was present in just 2 cases, and only 1 of these patients also had more than 50% central events in the diagnostic night.

**Conclusion:**

The population typically treated with ASV is entirely different from the study population in SERVE-HF, as nearly half of the patients treated with ASV showed a normal echocardiogram. Thus, the modified indication for ASV has little impact on the majority of treated patients. The current pathomechanistic hypothesis of central apnoea must be reviewed.

## Abstract (de)

**Ziel der Arbeit:**

Die SERVE-HF-Studie ergab keinen Vorteil der adaptiven Servoventilation (ASV) gegenüber der leitlinienbasierten medizinischen Therapie von Patienten mit symptomatischer Herzinsuffizienz, einer Ejektionsfraktion (EF) ≤45% und überwiegend zentralen Ereignissen (Apnoe-Hypopnoe-Index [AHI] >15/h). Weil die Gesamt- und die kardiovaskulär bedingte Mortalität in der ASV-Gruppe höher waren, wurden eine EF ≤45% in Kombination mit einem AHI 15/h, einem zentralen Apnoe-Hypopnoe-Index (CAHI)/AHI >50% und einem zentralen Apnoe-Index (CAI) >10/h als Kontraindikationen für die ASV aufgeführt. Ziel der vorliegenden Studie war es, die klinische Relevanz dieser Einschränkungen zu untersuchen.

**Methoden:**

Retrospektiv wurden die Daten von mit ASV behandelten Patienten ausgewertet, bei denen in der Nachsorge eine Echokardiographie zur Feststellung von Kontraindikationen für eine ASV erfolgt war.

**Ergebnisse:**

Bei 23 Patienten wurde eine Echokardiographie durchgeführt. In 10 Fällen war der Befund der Echokardiographie normal, eine linksventrikuläre Hypertrophie mit normaler EF wurde bei 8 Patienten festgestellt, eine EF von 45–50% bestand in 2 Fällen und eine valvuläre Aortenstenose (Grad II) mit normaler EF in einem Fall. Eine EF <45% bestand in nur 2 Fällen, und lediglich einer dieser Patienten wies mehr als 50% zentrale Ereignisse in der diagnostisch ausgewerteten Nacht auf.

**Schlussfolgerung:**

Die gewöhnlich mit ASV behandelte Population stellt sich völlig anders dar als die Studienpopulation in der SERVE-HF-Studie, da fast die Hälfte der Patienten mit ASV-Therapie einen normalen Echokardiographiebefund aufwies. Daher hat die modifizierte Indikation für eine ASV nur geringen Einfluss auf die Mehrzahl der behandelten Patienten. Die aktuelle Hypothese zu den Pathomechanismen der zentralen Apnoe muss überprüft werden.

## Introduction

Central sleep apnoea is associated with poor prognosis in chronic heart failure (HF), with some studies suggesting it to be the best predictor for mortality [1]. A recent study of more than 6500 patients with systolic HF in Germany reported strong associations between sleep-disordered breathing (SDB; either OSA or CSA) and obesity, male sex, atrial fibrillation, age and worse left ventricular systolic function [2]. In addition to haemodynamic compromise, central apnoeas cause sympathetic nerve activity (SNA) in HF [3] by deactivating pulmonary stretch receptors as well as hypoxia-/hypercapnia-driven stimulation of chemoreceptors. Apnoea-associated arousals raise the central sympathetic outflow whereas vagal outflow is reduced [3].

Patients with HF and concomitant CA show an increased heart rate and decreased variability in blood pressure and heart rate compared to HF patients without CA [4], as well as increased serum and urinary catecholamine levels [5] and increased muscle sympathetic nerve activation (MSNA) [6, 7]. The increase in SNA by CA causes arrhythmias and HF-related death, especially daytime CA-CSR [1, 8]. Specifically, CSA has been associated with ventricular tachycardia (VT) occurrence, irrespective of sleep/wake status in HFrEF patients, and independently predicted VT occurrence [9].

In contrast, other studies failed to show that CAs yield any prognostic information after correcting for underlying HF severity [10], which could suggest that CSA is no more than a reflection of poor cardiac function and sympathetic overdrive in systolic HF.

Furthermore, persistent central sleep apnoea/Hunter–Cheyne–Stokes breathing despite the best use of guideline-based therapy in heart failure patients with reduced ejection fraction could be a compensatory mechanism that should not be suppressed [11, 12]. By this hypothesis, which is mainly held by Naughton, the compensatory aspects of CSA-HCSB offset the oedematous lungs with restricted lung volumes, exhaustion due to an increased respiratory effort, bronchial wheeze due to the oedematous small airways, the desire to sit upright and difficulty sleeping, all of which are complications of HF. The benefits of CSA-HCSV could include enhanced forward cardiac output resulting in alkalosis, which has been shown to protect the failing, hypoxic heart from decompensation. Finally, CSA-HCSV increases oxygen stores. Nevertheless, the hypothesis that CSA-HCSV is protective remains very controversial [13].

The interaction between the heart and respiratory function is complex. CSA may not simply reflect poor cardiac function but even more, treatment of HF may itself improve SDB, as some evidence has suggested that CRT improved cardiac function and reduced the AHI [14]. SDB may also be reduced after successful percutaneous mitral valve repair [15]. Other studies generated similar conclusions that reliably showed statistical significance but revealed only a minor effect of unknown clinical significance [16].

Regarding treatment algorithms, treatment of coexisting OSA by continuous positive airway pressure (CPAP) in medically treated patients with HF reduces systolic blood pressure and improves left ventricular systolic function [17]. The improvement in NYHA classification and EF by CPAP compared to standard medical treatment has been confirmed by a recent meta-analysis [18].

Until 2015, patients with central sleep apnoea were often treated with adaptive servoventilation (ASV), a noninvasive ventilator therapy. ASV provides a servocontrolled positive expiratory pressure and inspiratory pressure support based on estimating the minute ventilation with which to target support. The pressure is automatically adjusted to stabilize and reduce ventilation. In the case of apnoea, the ventilator maintains ventilation while avoiding hyperventilation. A recent meta-analysis showed that CPAP and ASV are effective in improving LVEF in patients with heart failure as well as CSA/CSR in a clinically relevant manner, whereas nocturnal O_2_ is not. There were no differences between CPAP and ASV in terms of beneficial effects on cardiac function [19, 20].

The SERVE-HF [21] study sought to investigate the long-term effects of ASV in selected patients with reduced EF and dominant central apnoea. The primary and secondary endpoints of the study were evaluated as combined endpoints: Primary endpoint was the first event of the composite of death from any cause, a lifesaving cardiovascular intervention or an unplanned hospitalisation for worsening chronic heart failure. Subsequent secondary endpoints were defined as follows: The first secondary endpoint was defined as cardiovascular death, a lifesaving cardiovascular intervention or an unplanned hospitalisation for worsening chronic heart failure; the second secondary endpoint was defined as death from any cause, a lifesaving cardiovascular intervention or an unplanned hospitalisation for any cause. Neither the primary nor either of the two secondary endpoints showed any difference between ASV and guideline-based medical treatment.

Nevertheless, after analysing individual factors, a difference in all-cause as well as cardiovascular mortality was identified between the groups. In reaction, 6 months before the study was released, the main sponsor and manufacturer of ASV devices (ResMed Ltd, 1 Elizabeth Macarthur Drive, Bella Vista 2153, Australia) published new contraindications for ASV therapy: EF ≤45% in combination with >AHI 15/h, CAHI/AHI >50% and CAI >10/h.

As a consequence of the study findings, we recalled patients treated with ASV and performed echocardiography. Those patients with an EF ≤45% were also reanalysed for incidence of central events in their diagnostic polysomnography.

The hypothesis of our study was that the patients recruited in the SERVE HF trial represent only a minor group of patients in which the use of ASV is contraindicated compared to the patients in whom ASV could still be used.

## Materials and methods

### Patients

Following the SERVE-HF safety warning, 23 patients who had been treated with ASV and followed-up in Wuppertal were contacted by phone and/or mail to receive echocardiography and reanalyse their diagnostic polysomnography. The indication for ASV therapy had been Cheyne–Stokes respiration (CSR).

### Design

This study is a retrospective analysis of collected data, prompted by the new recommendations for ASV therapy. For this reason, no approval by an ethical committee was needed.

### Analysis

We analysed the data on the SERVE-HF contraindications against ASV.

## Results

Echocardiography was conducted in the 23 patients who had received ASV therapy. Anthropometric data are presented in Table 1. In 10 cases the echocardiogram was normal, in 8 patients a left ventricular hypertrophy with normal EF was found (all patients with HFpEF according to 2016 ESC guidelines), 2 cases showed an EF 45–50% and a valvular aortic stenosis (grade II) with normal EF was found in 1 case. An EF <45% was found in only 2 cases, and only 1 of these 2 patients also had more than 50% central events in the diagnostic night (Table 2).

**Table 1:** Demographic data

Mean age (years)	66
≤60 years (*n*)	10
61–70 years (*n*)	3
71–80 years (*n*)	6
>80 years (*n*)	4
Male sex (*n*; %)	21 (91)
Body mass index^a^ (kg/m^2^; min.–max.)	32 (22–47)
Arterial hypertension—n (*n*)	9
Diabetes mellitus—n (*n*)	5
Coronary heart disease—n (*n*)	5

^a^Missing data: 9

**Table 2:** Echocardiographic findings and central events

*n* = 23	Echocardiography	Patients with central events >50% (*n*)
10	Normal findings	0
8	Left ventricular hypertrophy	0
2	EF 46–50%	0
1	Aortic valve stenosis II	0
2	EF ≤45%	1

*EF* ejection fraction

## Discussion

Analysis of our cohort revealed only one patient meeting the inclusion criteria of the SERVE-HF study who was now contraindicated for ASV therapy. Most of the investigated patients had either a normal echocardiogram or left ventricular hypertrophy (HFpEF).

The SERVE-HF study results did not show any difference between the two groups in terms of the primary and secondary endpoints in patients with an EF ≤45%. Nevertheless, death from any cause (hazard ratio, HR, 1.06–1.55) and cardiovascular death (HR 1.09–1.65) were higher in the ASV group. These results led to new recommendations and contraindications for ASV therapy.

Remarkably, a reduced EF was rarely diagnosed in our patients. Therefore, the group of patients treated with ASV in our clinical cohort differed entirely from the group included in the SERVE-HF study. This prompts several questions: Did we treat the correct patients with ASV? If yes, what is the “real” pathophysiology of central sleep apnoea (CSA) if systolic left ventricular function is normal in most subjects?

Among patients with congestive heart failure (CHF) with NYHA II or higher, 50–75% (40% central, 36% obstructive) show sleep-disordered breathing (SDB) [22, 23]. The prevalence increases with age, male gender, NYHA class, nycturia, six-minute walking test, lower oxygen uptake, lower blood pressure, reduced LV-EF and enlarged atrial dimensions [22].

A reduced cardiac output leading to circulatory delay is a common explanation for the emergence of CSA. Heightened ventilatory response to PCO_2_ resulting in hypocapnia is also thought to contribute to CSA [24].

**Fig. 1:**
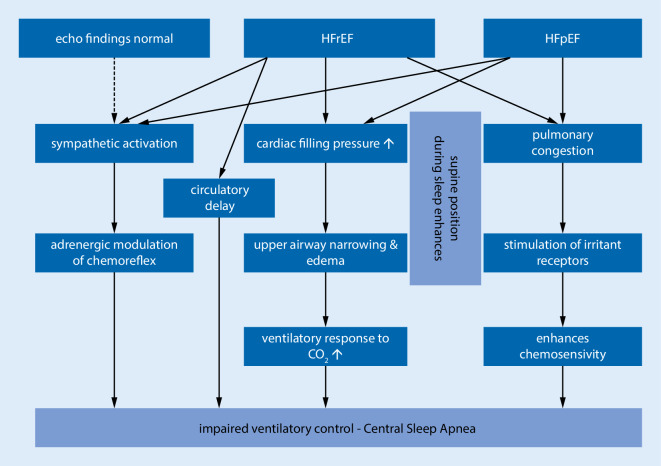
Direct mechanical effects and loss of neuromuscular control mechanisms together contribute to the pathophysiology of central sleep apnoea (CSA). Several risk factors associated with CSA are not explained by these pathways (e.g., male gender, age). Sympathetic activation seems to be the only mechanism that may contribute to CSA in patients with normal echocardiographic findings. It is also the only mechanism that is shared by the three different heart conditions. *HFrEF* heart failure with reduced ejection fraction, *HFpEF* heart failure with preserved ejection fraction

Moving from the upright to the supine position worsens CSA [25] and causes an increase in cardiac filling pressure [26]. The latter is of particular interest because an increase in cardiac filling pressure and dilatation of the left atrium can be observed in LV hypertrophy, which was common in our population. Indeed, the association between heart failure with preserved ejection fraction (HFpEF) and CSA has been described previously: In 878 patients with symptomatic heart failure assessed for systolic LV function, 366 patients showed a normal EF (valvular disease in 108 cases) and ~70% showed SDB (30% CSA, 40% OSA) [27]. Given this study, it seems that the role of CSA in HFpEF is often ignored in the conversation about CSA, and thus our findings are quite novel. The fact that pulmonary arterial wedge pressure (PAWP) also correlates with CSA incidence suggests another explanation for CSA, i.e., that pulmonary congestion stimulates the pulmonary vagal irritant receptors, which enhances chemosensitivity, leading to hyperventilation and respiratory instability [5, 26, 28, 29]. This is supported by the fact that diuretics lead to a significant decrease in AHI among patients with diastolic heart failure by reducing upper airway oedema and pulmonary congestion [30]. The pathophysiology of CSA is summarized in Fig. 1.

There may be other mechanisms by which CHF leads to CSA. CSA may also be observed following stroke, but there is no relation to the location or type of stroke [31]. Opioid use is also associated with the development of central sleep apnoea in general and CSR in particular. The prevalence of CSA in chronic opioid treatment is ~25%, depending on doses (especially a morphine equivalent daily dosage, MEDD, >200 mg/day) and normal or low body mass index [32, 33]. In addition, periodic breathing occurs at high altitude due to an imbalance in the negative feedback loop of the ventilation drive [34]. This may help keep the level of oxygen saturation high. Considering all the reasons for CSA development together, we did not observe any additional reasons for CSA in our cohort.

With our results, there are several additional questions that must be addressed to the authors of the SERVE-HF study. If a markedly reduced EF is an exception rather than the rule, then it would be important to know how many patients were screened in the SERVE-HF study to include the final 1325 patients. Given the data of our small sample that only 4% of patients met the inclusion criteria of SERVE-HF, a rough calculation suggests that 33,125 patients would need to have been screened. Furthermore, patients were randomised to receive optimal medical treatment alone or optimal medical treatment plus ASV at a 1:1 ratio [35]. However, neither the final study publication including supplementary online material nor the published study design paper contained any details on medical doses or changes in medical treatment. Therefore, it remains unclear whether the two study groups in SERVE-HF differed regarding medical treatment or if the medical treatment was indeed optimal (follow-up time 24–84 months), which has been identified as a problem in earlier studies [36, 37].

Recent study results indicate that ASV therapy does not decrease sympathetic drive in patients with systolic heart failure. In CSA patients with normal cardiac function (ICSA), similar pressure levels of PAP but not ASV favourably altered sympathovagal balance [38]. The clinical importance of this difference remains to be determined. A recent meta-analysis did not find meaningful differences between CPAP and ASV therapy regarding cardiac endpoints [18].

## Limitations

There are several limitations of our study that must be noted. First, the demographic and clinical characteristics our cohort are incomplete due to the retrospective nature of our study. Nevertheless, the aim of our study was not to analyse the reasons for CSA in our cohort, but rather to identify patients with exclusion criteria for ASV and to estimate the incidence of the combination of reduced EF and central apnoea >50% in patients treated with ASV. Second, the sample size of our cohort is small. The intention of our analysis was to obtain a first impression of the incidence rather than to perform a prospective analysis.

## Conclusion

Our preliminary data indicate that the population typically treated with ASV is quite different from the study population in SERVE-HF, as nearly half of the patients treated with ASV showed a normal echocardiogram. The altered indication for ASV following publication of the SERVE-HF trial seems to have relatively little impact on the majority of treated patients.

## Abbreviations

AHI: Apnoea–hypopnea index
ASV: Adaptive servoventilation
CA: Central apnoea
CAI: Central apnoea index
CAI/AHI: Central apnoea index/apnoea–hypopnea index
CHF: Congestive heart failure
CPAP: Continuous positive airway pressure
CRT: Cardiac resynchronization therapy
CSA: Central sleep apnoea
CSR: Cheyne–Stokes respiration
CVD: Cardiovascular disease
EF: Ejection fraction
HFpEF: Heart failure with preserved ejection fraction
HFrEF: Heart failure with reduced ejection fraction
HCSB: Hunter–Cheyne–Stokes breathing
HCSV: Hunter–Cheyne–Stokes ventilation
MACE: Major adverse cardiovascular event
MEDD: Morphine equivalent daily dosage
MSNA: Muscle sympathetic nerve activation
NYHA: New York Heart Association
OSA: Obstructive sleep apnoea
SDB: Sleep-disordered breathing
SNA: Sympathetic nerve activation
VT: Ventricular tachycardia
